# Introducing Lu-1, a Novel *Lactobacillus jensenii* Phage Abundant in the Urogenital Tract

**DOI:** 10.1371/journal.pone.0234159

**Published:** 2020-06-11

**Authors:** Taylor Miller-Ensminger, Rita Mormando, Laura Maskeri, Jason W. Shapiro, Alan J. Wolfe, Catherine Putonti

**Affiliations:** 1 Bioinformatics Program, Loyola University Chicago, Chicago, IL, United States of America; 2 Department of Biology, Loyola University Chicago, Chicago, IL, United States of America; 3 Department of Microbiology and Immunology, Stritch School of Medicine, Loyola University Chicago, Maywood, IL, United States of America; 4 Department of Computer Science, Loyola University Chicago, Chicago, IL, United States of America; State Key Laboratory for Diagnosis and Treatment of Infectious Diseases, CHINA

## Abstract

Bacteriophages (phages) play a key role in shaping microbial communities, including those of the human body. Phages are abundant members of the urogenital tract, most often persisting through the lysogenic life cycle as prophages integrated within the genomes of their bacterial hosts. While numerous studies of the urogenital microbiota have focused on the most abundant bacterial member of this niche–*Lactobacillus* species–very little is known about *Lactobacillus* phages. Focusing on *Lactobacillus jensenii* strains from the urinary tract, we identified numerous prophages related to the previously characterized Lv-1 phage from a vaginal *L*. *jensenii* strain. Furthermore, we identified a new *L*. *jensenii* phage, Lu-1. Evidence suggests that both phages are abundant within the urogenital tract. CRISPR spacer sequences matching to Lv-1 and Lu-1 prophages were identified. While first detected in urinary isolates, the Lu-1 phage was also discovered in *L*. *jensenii* isolates from vaginal and perineal swabs, and both phages were found in metagenomic data sets. The prevalence of these phages in the isolates suggests that both phages are active members of the urogenital microbiota.

## Introduction

Lactobacilli are a prominent member of the healthy female urogenital tract [1]. 16S rRNA gene sequencing and shotgun whole genome sequencing of the vaginal and urinary tract microbiota have identified the same species in both niches, suggesting that the two microbiota are in fact interconnected [2–4]. The healthy vaginal microbiota is often dominated by one of four *Lactobacillus* species: *Lactobacillus crispatus*, *L*. *iners*, *L*. *gasseri*, or *L*. *jensenii* [5, 6]. These species have been found to play a key protective role in the vagina [7–14]. For example, *Lactobacillus* species can inhibit the growth of *Escherichia coli*, including uropathogenic strains [15–17]. A decreased abundance of lactobacilli within the vagina has been associated with decreased conception rates and increased rates of early pregnancy loss [18], as well as bacterial vaginosis (BV) [19]. Lactobacilli are also abundant within the urinary microbiota of women with and without lower urinary tract symptoms (LUTS) [20–27]. While *L*. *gasseri* is more frequently detected in the urinary microbiota of women with urgency urinary incontinence, *L*. *crispatus* is more frequently found within the urinary microbiota of women without LUTS [20]. Prior research has shown that *L*. *jensenii* is protective against *E*. *coli* and urinary tract infection (UTI) development [16], and that *L*. *iners* protects against post-operative UTI [28].

In addition to the bacterial members of the human microbiota, bacteriophages (viruses that infect bacteria) are abundant [29]. Moreover, bacteriophages (phages) play a role in microbiota stability and human health (see review [30]). Phages within the gut and oral cavity have been associated with gastrointestinal and periodontal disease, respectively [31–33]. Similarly, it has been postulated that BV is the result of phage predation in the vaginal microbiota [34]. It is important to note, however, that in contrast to other areas of the human body, the phage communities within the urogenital tract are understudied. To date, very few viral metagenome studies have been conducted for the urinary tract, and we have only begun cataloging the phages present within this niche (see recent review [35]). Early evidence suggests that phages replicate within the urogenital tract microbiota mainly through the lysogenic life cycle–the phage’s genome (prophage) is integrated within the genome of their bacterial host and passed to subsequent generations. Previous research found that vaginal lactobacilli are frequently lysogens, harboring several prophages within their genomes [36–41]. Recently we found that bacteria of the bladder microbiota also are often lysogens [42]. Despite the observed abundance of *Lactobacillus* lysogens within the urogenital tract [36–42], these phages are very poorly understood. To date, only a few *Lactobacillus* phages from the vaginal microbiota have been isolated [37–39] and no phages from the urinary microbiota have been isolated. Furthermore, only one *Lactobacillus* phage has been sequenced: *L*. *jensenii* phage Lv-1, isolated from the vagina [43,44]. While some of the prophage sequences previously identified in urinary lysogens resemble the Lv-1 genome, these are only partial sequence similarities; most of these prophage sequences exhibit no sequence homology to any characterized phage [42].

In contrast to lactobacilli of the vaginal microbiota, relatively few *Lactobacillus* strains have been sequenced from other areas of the urogenital tract. We recently sequenced urinary lactobacilli, representative of eight different species of this genus [4]. We subsequently sequenced an additional 11 *L*. *jensenii* isolates [45]. Here, we present the results of our examination of these 11 *L*. *jensenii* genomes for prophages, finding multiple instances of the previously characterized Lv-1 phage. We also identified a new phage group, which we have named *Lactobacillus* phage Lu-1 for *L**actobacillus* urogenital phage. We mined publicly available microbiome data sets in addition to screening 63 (unsequenced) isolates from the urogenital tract via PCR amplification, searching for Lv-1 and Lu-1, and found an abundance of both phages.

## Materials & methods

### *L*. *jensenii* strains

Seventy-four *L*. *jensenii* strains were isolated and cultured using the Expanded Quantitative Urinary Culture (EQUC) protocol [46] as part of prior IRB-approved studies [4, 20–22, 24, 46, 47]. These isolates were identified by Matrix-Assisted Laser Desorption/Ionization-Time Of Flight (MALDI-TOF) mass spectrometry. Strains include those isolated from vaginal swabs, perineal swabs, catheterized urine, and voided urine (**Table 1**). Vaginal and perineal samples were collected according to standard clinical practice. Swabs were collected and stored using the BD Liquid Amies Elution Swab Collection/Transport system (BD 220245). The EQUC protocol [46] was used to isolate individual strains. This protocol was used as previously described for the urine samples; vaginal and perineal swabs were similarly processed using EQUC with the exception that 10 μL of the Liquid Amies Elution swab was used for plating. Eleven of the catheterized urine isolates were sequenced previously [45]. The genomes for the remaining isolates included in this study have not been sequenced.

**Table 1:** Isolation source of 74 urogenital *L*. *jensenii* strains.

Isolate Source	Number of Isolates
Vaginal swab	6
Perineal swab	2
Catheterized urine	40
Voided urine	26

Catheterized urine isolates include the 11 strains previously sequenced [45]. The genomes of the remaining 63 isolates have not been sequenced.

### Identifying *L*. *jensenii* prophages

Recently, we sequenced 11 *L*. *jensenii* isolates from catheterized urine samples from women and assembled their draft genomes [45]. Genome assemblies were screened for the presence of prophage sequences using the web-based tool PHASTER [48]. Predicted prophage regions were annotated using RAST [49]. These functional annotations were further refined manually via blastp queries to the nr database. We then aligned the predicted prophage sequences using progressiveMauve algorithm v1.1.1 [50] in Geneious Prime (Biomatters Ltd., Auckland, New Zealand).

### Identifying CRISPR spacer arrays

CRISPR spacer arrays were identified within the 11 *L*. *jensenii* genomes from catheterized urine samples [45] using CRISPRFinder [51]. Matches between spacer sequences and PHASTER predicted prophage sequences were performed by aligning sequences via local blastn. Only blast hits producing complete and identical matches were recorded (query coverage = 100%; identity = 100%).

### Screening metagenomes for *L*. *jensenii* phages

The Lv-1 RefSeq sequence (GenBank: NC_011801) and the Lu-1 sequence for prophage 1307_1 were used to query publicly available unassembled genomes and metagenomes in NCBI’s SRA database via www.searchsra.org. For each SRA record producing hits to one of the two sequences, we calculated the coverage for each nucleotide in the query (Lv-1 or Lu-1) sequence using bbmap v34 (https://sourceforge.net/projects/bbmap/); the scripts bbwrap.sh and pileup.sh were used to map trimmed reads to the assemblies and compute average genome coverage. Mapping was performed using the pileup.sh script in bbmap. Default parameters were used including a minimum alignment identity of 76%, requiring 22 consecutive identical matches, and a maximum indel value of 80. Coverage output files were parsed using Python. The sample source for each SRA record was retrieved from NCBI’s BioSample database.

### Screening lysogens for prophages

The prophage sequences most similar to Lv-1 or Lu-1 were used to design primers. Four sets of primers were designed using Primer3 [52] to identify different conserved areas that are unique to either the Lv-1 or Lu-1 sequences. These primer pairs are listed in **Table 2**. To ensure that the primer pairs designed would only amplify the Lv-1 or Lu-1 sequence, each was queried against the complete nr/nt database via blastn. All hits with 70% sequence identity were considered possible mishybridizations. The specificity of the primer pairs was thus confirmed to be specific to the intended target. Primers were synthesized by Eurofins Scientific (Louisville, KY). Freezer stocks (-80°C) of each clinical *L*. *jensenii* isolate were streaked on a Columbia CNA agar plate with 5% sheep blood (BD 221353) and incubated at 35°C in 5% CO_2_ for 48 hours. Colonies were selected from each plate to test the presence of Lv-1 and Lu-1 unique regions. PCRs were conducted using PCR Master Mix (Promega M7502) and the following conditions: 95°C for 3 m; 30 cycles of: 95°C for 30 s, 54°C for 30 s, and 72°C for 1 m; 72°C for 5 m. PCR reactions were then run through a 1.2% agarose gel.

**Table 2:** PCR primers designed to amplify conserved sequences within prophages identified in 11 *L*. *jensenii* genomes [45].

Primer	Primer Sequences	Amplicon Length (bp)	Targeted Coding Region
Lv1	5’-AGG CGC AAG GTG AAG TAG-3’ and 5’-TCA ACA CGT TGC TTC TGG-3’	409	Lv-1_gp10 Tape measure protein
Lu1.1	5’-CGC ATA TTG TGC TGC TTG-3’ and 5’-TTC GTC AAG GTG TTC GTG-3’	355	Hypothetical protein
Lu1.2	5’-TTC GGC TCC TCA ACA ATC-3’ and 5’-TCA CCA CCA ACT ACA CCT TG-3’	437	Hypothetical protein
Lu1.3	5’-CGA ACA ACC AGC TTA GCC-3’ and 5’-CTT GCG TTG TTG CAC TTC-3’	406	Hypothetical protein

### Induction and screening for lytic phages

*L*. *jensenii* UMB1307 was selected to test for phages in the lytic cycle. A culture of the bacterium was grown by inoculating 5 mL of MRS medium, supplemented with 0.1% TWEEN® 80 (Sigma-Aldrich). Inductions were performed following published protocols [53, 54]. Briefly, cultures were grown overnight at 35°C with 5% CO_2_. 1 mL of the overnight culture was centrifuged at 10,000g for two minutes. The supernatant was filtered using a 0.22 um filter. DNase (OPTIZYME DNase I; Fisher BioReagents) was used to remove any remaining bacterial DNA. 50 ul of overnight culture was transferred to 5 mL of fresh MRS + tween medium and left to grow in the previous described conditions for two hours. This was done for 6 cultures, one control and 5 experimental. Mitomycin C was added to each experimental culture at different final concentrations: 0.1ug/mL, 0.2ug/mL, 0.3ug/mL, 0.4ug/mL, and 0.5ug/mL. The cultures were left to grow for overnight in the previously described conditions. Filtered and DNase-treated supernatants were then prepared as described above. PCR amplification, using the primers listed in **Table 2**, was used to check for the presence of phage in the filtrate before and after Mitomycin C treatment.

We also tried to induce the Lv-1 and Lu-1 phages via experimental evolution using all 8 of the strains harboring these prophages (UMB0034, UMB0037, UMB0055, UMB0732, UMB1165, UMB1303, UMB1307, and UMB1355). For each strain, 1 mL of MRS medium, supplemented with 0.1% TWEEN® 80, was inoculated with a single colony and grown overnight at 35°C with 5% CO_2_. The culture was then centrifuged at 10,000g for two minutes and the supernatant was removed and stored at 4°C. The bacterial pellet was resuspended in 1 mL fresh MRS + tween medium, vortexed gently, and then grown overnight again at 35°C with 5% CO_2_. This process was repeated for 5 days after which supernatants were filtered using a 0.22 um filter. DNase (OPTIZYME DNase I; Fisher BioReagents) was used to remove any remaining bacterial DNA. PCR amplification, using the primers listed in **Table 2**, was used to check for the presence of phage in the filtrate.

## Results

### Bioinformatic discovery of *L*. *jensenii* phages

The web-based PHASTER tool was used to predict prophages within the 11 urinary *L*. *jensenii* genomes. PHASTER identified 21 putative prophage regions; 11 were predicted as being “intact,” 3 as “questionable,” and 7 as “incomplete” prophage sequences. All of the 11 *L*. *jensenii* strains contained a predicted prophage sequence. PHASTER identified homologies to several different *Lactobacillus* phages, including *Lactobacillus* phage PLE2 (host = *L*. *casei* BL23), *Lactobacillus* phage LfeSau (host = *L*. *fermentum*), *Lactobacillus* phage Lj928 (host = *L*. *johnsonii*), *Lactobacillus* phage jlb1 (host = *L*. *gasseri* ADH), and *Lactobacillus* phage Lv-1 (host = *L*. *jensenii*) as well as *Bacillus* phage vB_BanS_Tsamsa (host = *B*. *anthracis*) (**S1 Table**). All of the intact predicted prophages exhibited the greatest sequence similarity to *L*. *jensenii* phage Lv-1. Three incomplete prophages also were most similar to the Lv-1 genome. In total, eight of the 11 urinary *L*. *jensenii* genomes contained at least one prophage sequence related to Lv-1.

Performing a blastn search of the Lv-1 genome sequence (GenBank: NC_011801) to NCBI’s nr/nt database returned homology to a single record–*L*. *jensenii* SNUV360 (CP018809), a bacterial isolate from the vagina [55]. Repeating this search against the WGS database, restricting the search to records identified as *L*. *jensenii*, identified five additional *L*. *jensenii* strains harboring the Lv-1 phage: IM18-1 (AZNN), IM18-3 (AZNN), SJ-7A-US (ACQD), 269–3 (ACOY), and 1153 (ABWG); while the source of the two IM18 strains is unknown, the other three were isolated from the vaginal microbiota as part of the Human Microbiome Project [56].

**Fig 1:**
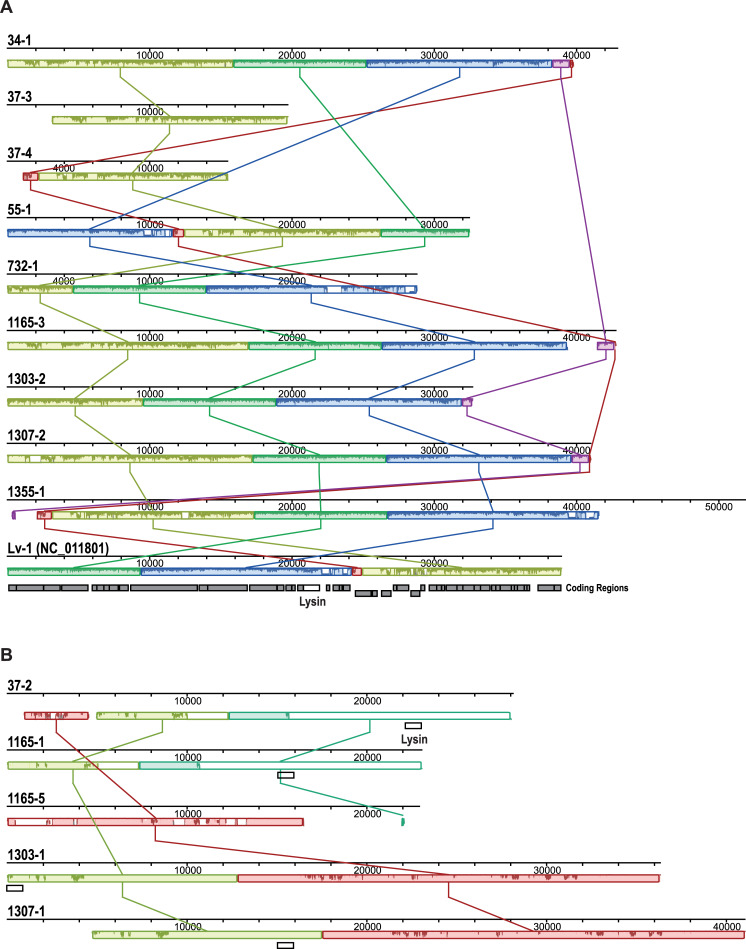
Alignments of 14 prophage sequences from eight sequenced *L*. *jensenii* genomes isolated from catheterized urine. (A) Lv-1 family of prophages, including the Lv-1 annotated genome (GenBank: NC_011801). (B) Lu-1 family of prophages. Individual prophages are named according to the strain from which they were identified.

The 14 putative prophages homologous to Lv-1 fall into two distinct groups (**Fig 1**). The first group most closely resembles the published Lv-1 phage genome (GenBank: NC_011801) (**Fig 1A**). The second group, which we have named Lu-1 for *L**actobacillus* urogenital phage, has very little sequence homology to the Lv-1 genome (**Fig 1B**). The blastn query of a representative Lu-1 sequence (prophage 1303–1) to the Lv-1 GenBank sequence reveals only 3% query coverage and 72.3% sequence identity between the two; regions of homology include Lv-1 annotated lysin (Lv-1_gp18) and single stranded DNA binding protein (Lv-1_gp36).

**Fig 2:**
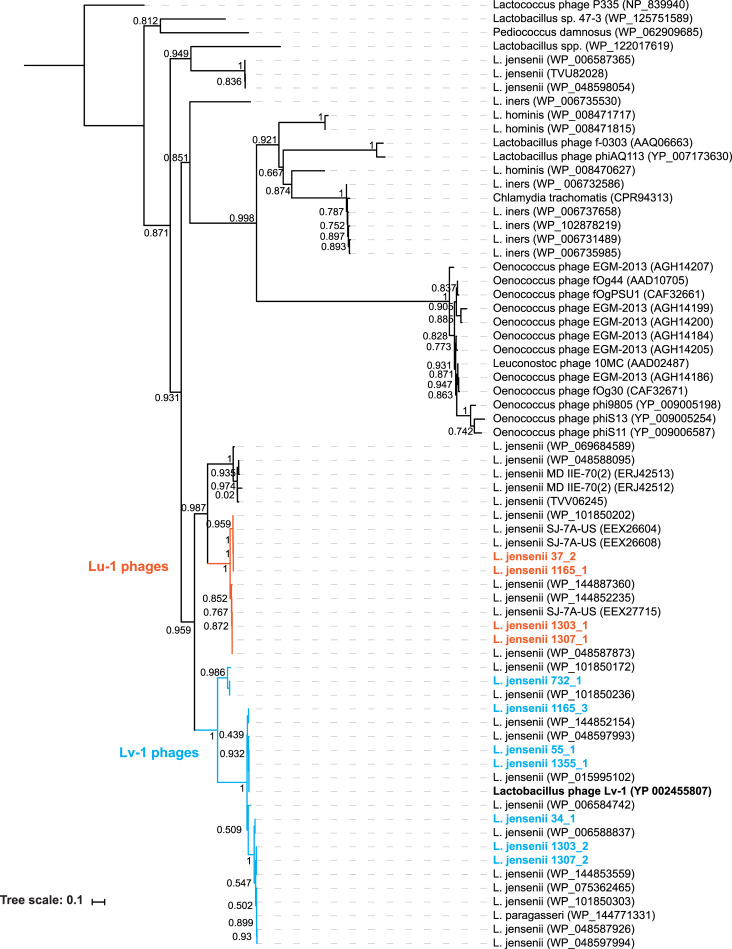
Phylogenetic tree of lysin amino acid sequences containing the GH25 conserved domain of Lv-1 (blue) and Lu-1 (orange) prophages. Branch likelihoods are shown.

As **Fig 1A** shows, the Lv-1 group of phages is highly conserved. The two sequences from UMB0037 are only partial sequences and were identified by PHASTER as incomplete. We first confirmed that these two incomplete phage sequences are two distinctly different sequences; the aligned nucleotide sequences of prophage 37–3 and 37–4 have only 59.49% sequence identity. Second, the two incomplete phage sequences are in fact partial sequences, not artifacts of assembly; we remapped the raw sequencing reads to the GenBank Lv-1 phage genome sequence. As **Fig 1B** illustrates, the Lu-1 group is distinct from the GenBank Lv-1 genome. The Lv-1 lysin coding region (Lv-1_gp18; GeneID: 7262053) is found in prophages 37–2, 1165–1, 1303–1, and 1307–1. This lysin coding sequence of the Lv-1 and Lu-1 phages shares a protein domain, the glycosyl hydrolase family 25 (GH25_muramidase). We queried the Lv-1 and Lu-1 lysin amino acid sequence against the GenBank nr protein database using the blastp algorithm with default parameters. We found >50 instances of the GH25-muramidase domain (identity > 35%) within *Lactobacillus* genomes and Lactobacillaceae-infecting phages (**Fig 2**). However, the Lv-1 and Lu-1 phage lysins have different C-termini (~170 aa), and these two C-terminal sequences do not resemble other proteins included in **Fig 2**. While the prophage sequence 1165–5 does not include the Lv-1 lysin coding region, it does encode for a homolog to the Lv-1 ssDNA binding protein (Lv-1_gp36; GeneID: 7262071). The 1165–5 prophage was also identified by PHASTER as incomplete.

As the Lu-1 group of phages exhibited very little sequence homology to characterized annotated phage or prophage sequences, we annotated these sequences by RAST [49], but they were also annotated by PGAP when the complete genomes were deposited in GenBank [45]. In addition, we queried each RAST-predicted open reading frame for the Lu-1 group sequences against the nr protein database via blastp and the nr/nt database via tblastn in an effort to improve our annotations (**S2 Table**). For most of the predicted open reading frames, the only blast hit with an E-value>1 was to the *L*. *jensenii* host genome record. Hallmark phage genes were of exception, although several exhibited only modest sequence homology. Gene annotations included phage capsid, scaffold, tape measure, terminase (small and large subunits), portal, single-stranded DNA-binding, holin, recombinase, and antirepressor, as well as the more generic “phage protein.” This manual curation also found the cI repressor protein encoded within both the predicted prophage sequences 1303_1 and 1307_1. Amino acid sequences for Lu-1 functionally annotated proteins were queried against the nr/nt database via tblastn; we found only a few protein hits. The Lv-1 and Lu-1 phages encode for different terminase, portal, capsid, scaffold, and tape measure proteins.

**Fig 3:**
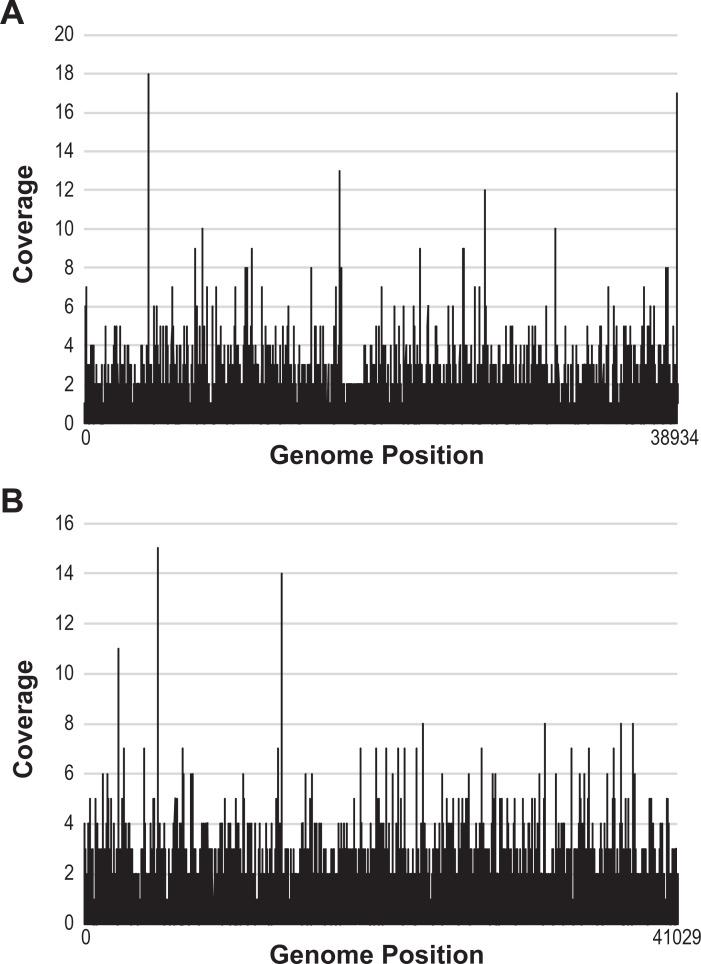
Lv-1 and Lu-1 detection in metagenome. Distribution of reads representative of the (A) Lv-1 and (B) Lu-1 genomes (GenBank: NC_011801 and the sequence for prophage 1307_1, respectively) from the US Gulf of Mexico marine sediment metagenome (SRR2090130).

Given the dearth of information about *L*. *jensenii* infecting phages, we were curious if the Lv-1 and Lu-1 phages were perhaps common within the human microbiota. We next searched for Lv-1 and Lu-1 phage sequences in unassembled metagenomic datasets via searchsra.org, which mines publicly available reads (NCBI’s SRA database) for a given query sequence. The complete Lv-1 and Lu-1 prophage sequences were queried against these data sets identifying hits from 1797 metagenomes each. The majority of these hits were hits to a single gene. Data sets covering the query sequence >40% were investigated further, revealing 16 Lv-1-like and 19 Lu-1-like sequences from sediment, marine, and rumen samples (**S3 Table**). Further inspection of this subset of metagenomes found that the metagenomic reads represented genes across the genome (**Fig 3**), i.e. reads were not exclusively mapping to a subset of genes. This distribution suggests that relatives of Lv-1 and Lu-1, or at the very least the genes encoded by these two phages, are likely present within these samples.

Given the prevalence of Lv-1 within these 11 bladder isolates, we next examined their genomes for the CRISPR/Cas system, looking for evidence of past infections of these strains by Lv-1 and Lu-1 phages. While CRISPR/Cas adaptive immunity is present in many *Lactobacillus* species, it is not necessarily present in all strains [57]. Six of the 11 *L*. *jensenii* genomes included the CRISPR/Cas system, and five of these genomes included at least one spacer sequence that was identical to a subsequence of the Lv-1 or Lu-1 prophage sequences. Three urinary *L*. *jensenii* strains that did not contain an Lv-1 or Lu-1 prophage–UMB7848, UMB8345, and UMB8489 –contained more than one spacer sequence to the Lv-1 genome (**Table 3**), suggesting that the spacer prohibited the integration of the phage within its genome. UMB7848 and UMB8489 also contain spacer sequences to the Lu-1 prophage sequence. Interestingly, UMB1165 and UMB0034, which are lysogens of the Lu-1 and Lv-1 prophages, respectively, also contained spacer sequences to the Lv-1 phage. However, their spacer sequences did not match to the Lv-1 prophage within their own genome.

**Table 3:** CRISPR spacer matches to Lv-1 and Lu-1 prophage sequences within the bladder *L*. *jensenii* strains.

Strains/ Spacer Array Position	Lv-1 Prophages									Lu-1 Prophages				Protein Function Targeted
	34–1	37–3	37–4	55–1	732–1	1165–3	1303–2	1307–2	1355–1	37–2	1165–1	1303–1	1307–1	
**UMB0034**														
*Spacer 3*			X	X					X					*Ci-like Repressor*
*Spacer 10*			X	X					X					*Ci-like repressor*
**UMB1165**														
*Spacer 3*			X	X					X					*Ci-like repressor*
**UMB7848**														
*Spacer 3*	X			X	X				X					*Terminase*, *large*
*Spacer 18*										X	X			*Hypothetical*
*Spacer 21*												X	X	*Hypothetical*
**UMB8345**														
*Spacer 5*	X						X	X						*Tail fiber*
*Spacer 6*	X				X									*Tape measure*
*Spacer 7*	X	X				X								*Noncoding*
*Spacer 10*									X					*Major head*
*Spacer 12*							X	X	X					*Major head*
**UMB8489**														
*Spacer 1*	X			X		X	X	X	X					*Holin*
*Spacer 5*										X	X			*Hypothetical*
*Spacer 8*					X	X			X					*Hypothetical*
*Spacer 10*										X	X			*Hypothetical*

Each spacer shown is a distinct sequence matching to the listed coding region.

### Lv-1 and Lu-1 phages are abundant in urogenital *L*. *jensenii* strains

To ascertain the prevalence of these two phages within the urogenital tract, we designed primers to target conserved regions of the Lv-1 and Lu-1 prophage sequences from the 11 urinary isolates. These primers were confirmed via exhaustive blast analyses to be specific to either the Lv-1 or Lu-1 prophage sequence (see Methods). Using these primers, we screened an additional 63 *L*. *jensenii* isolates that have not been sequenced. This includes 55 *L*. *jensenii* isolates from urine, two *L*. *jensenii* isolates from perineal swabs, and six *L*. *jensenii* isolates from vaginal swabs. Four primer pairs were designed (**Table 2**), one pair to detect Lv-1 and three pairs to detect different regions of the Lu-1 phage sequence. This screening identified an additional 10 Lv-1 prophages and 40 Lu-1 prophages within the isolates (**Table 4**). Both of the isolates from perineal swabs included one of these prophages. As for isolates from the other anatomical sites sampled, half of the samples included an Lv-1 and/or Lu-1 prophage. **S4 Table** lists the results for each *L*. *jensenii* isolate tested. The Lu-1 phage was detected in all of the niches tested. Nevertheless, we were not able to isolate the Lv-1 or Lu-1 prophages in the lytic cycle via standard induction protocols or experimental evolution assays (see Methods).

**Table 4:** Results of PCR screening for Lv-1 and Lu-1 prophages in urogenital tract isolates.

Isolate Source	Lv-1 only	Lu-1 only	Both
Vaginal swab (n = 6)	0	2	1
Perineal swab (n = 2)	0	2	0
Catheterized urine (n = 29)	3	14	4
Voided urine (n = 26)	0	15	2

## Discussion

Despite the prevalence of lactobacilli in the urogenital tract, very little is known about the phages that infect this genus. Sequencing of *Lactobacillus* strains from the urinary tract revealed that, like strains isolated from the vaginal microbiota [36–41], most urinary lactobacilli are lysogens [42]. As shown here, this also holds true for *L*. *jensenii* strains of the urinary tract. Eight of the 11 urinary *L*. *jensenii* genomes examined here were predicted to include prophages. The most abundant group of prophages resembled the *Lactobacillus* phage Lv-1. Further analysis of these sequences revealed that these bacteria in fact harbored two different phages: phages closely related to the previously characterized Lv-1 phage and a new phage presented here, *Lactobacillus* phage Lu-1 (**Fig 1**). The lysins encoded by Lv-1 and Lu-1 are unique to these two phages (**Fig 2**) [58]. Our screening of isolates from urinary tract samples, as well as vaginal and perineal swabs, showed that Lv-1 and Lu-1 are abundant within these microbiota (**Table 3**). Furthermore, mining of publicly available metagenome projects uncovered homologous sequences in soil, sediment, and marine samples (**S3 Table**); the literature provides no mention of *L*. *jensenii* within these environments.

Currently, there is debate as to whether the vaginal and urinary tract communities are distinct microbiota or if they are interconnected. 16S rRNA gene sequence studies have identified the same genera across the two sites [2, 3]. Whole genome sequencing of vaginal and urinary tract isolates of the same species from the same individual suggests that the two microbiota are connected [4]. If the bacterial constituents of the vaginal and urinary tract are interconnected, it would follow that the phage communities of these two niches are connected as well. The presence of both Lv-1 and Lu-1 in *L*. *jensenii* isolates from urine samples, perineal swabs, and vaginal swabs suggests that this is true (**Table 3**). Given the number of *L*. *jensenii* strains harboring one or more of these phages, we hypothesize that these phages are members of the core phage community of the urogenital tract. While core phage communities have been identified for other areas of the human body, e.g. the gut [59], no such community has yet to be identified within the urogenital tract as relatively few virome studies have been conducted [35, 60].

Analysis of the CRISPR spacer arrays within the 11 urinary *L*. *jensenii* strains found that the Lv-1 and Lu-1 phages have been repeatedly infecting this species. Several *L*. *jensenii* genomes included CRISPR spacers to Lv-1, Lu-1, or, in the case of UMB7848 and UMB8489, spacers to both phages. While our PCR-based screening for these two phages identified a much higher prevalence of Lu-1, only two of the 11 urinary *L*. *jensenii* strains, UMB7848 and UMB8489, included CRISPR spacers for the Lu-1 genome sequence (**Table 3**). Several of the spacer sequences were perfect matches to Lv-1 prophages found in other *L*. *jensenii* strains. For instance, the *L*. *jensenii* UMB1165 genome is both infected by an Lv-1 phage and includes a CRISPR spacer sequence for Lv-1, matching to the Lv-1 prophage of UMB0037, but not its own Lv-1 prophage sequence (**Table 3**). Mutations observed between the Lv-1 prophage sequences suggest multiple instances of escape mutants that evaded the *L*. *jensenii* CRISPR/Cas system. Several of the bacterial strains include multiple spacers to Lv-1. For instance, UMB8345 includes five spacers to the Lv-1 genome, suggesting that it has encountered multiple separate infections by the Lv-1 phage.

Furthermore, the CRISPR spacer arrays suggest that Lv-1 is not only persistently infecting *L*. *jensenii*, but has done so recently. In fact, all CRISPR arrays show the spacers for Lv-1 as relatively new additions (closer to the leader sequence upstream of the CRISPR spacer array) (**Table 3**). Isolate UMB8489 harbors an Lv-1 spacer in the most recent position (position 1), indicating a newer infection [61]. While the CRIPSR spacer analysis identifies recent Lv-1 infections, previous work described Lv-1 as a “defective” phage [44]. While the aforementioned study was able to successfully induce and image the Lv-1 phage, they were unable to infect *L*. *jensenii* strains isolated from the vagina, thus qualifying it as a defective phage [44]. Our annotations of the Lv-1 and Lu-1 prophage sequences indicated that several are intact, and likely viable.

Although we detected the Lv-1 and Lu-1 prophages in numerous strains (**Table 4**), our own attempts to induce the phages using Mitomycin C and experimental evolution yielded no results (see Methods). However, there are many other methods for phage induction and, as prior work has shown, varying concentrations of Mitomycin C applied at different times of the bacterial life cycle can have very different effects [62]. In the work of Martín *et al*. [43], Lv-1 was able to be induced with Mitomycin C at a concentration of 0.45 μg/ml, the authors were unable to produce plaques and thus labeled the phage as “defective” [43,44]. We tested a broader range of Mitomycin C concentrations, including 0.45 μg/ml, but were unable to detect the presence of the Lu-1 genome extra-cellularly via PCR. Although neither Lv-1 nor Lu-1 have yet to be characterized in the lytic cycle, CRISPR sequence analysis suggests that it is likely an active phage within the urinary tract microbiota. In the future, we will continue to test methods for induction on these urinary *L*. *jensenii* strains to ascertain if either phage can enter the lytic cycle.

*Lactobacillus* phages may play a role in modulating their community structure, and thus contributing to disease or protecting against pathogens. For instance, a *L*. *johnsonii* phage has been shown to protect against *Clostridium* in the gastrointestinal tract via production of its endolysin [63]. Lysins have been found to be an effective antimicrobial [64, 65]. The unique lysin encoded by the Lv-1 and Lu-1 phages may have similar antibacterial properties within the urogenital tract. While here we have focused on just two of the prophage sequences that have been identified in *L*. *jensenii* strains, there are other predicted *Lactobacillus* prophage sequences in the urogenital tract [37,38]. Analysis of CRISPR spacer sequences in *L*. *jensenii*, as well as other urogenital *Lactobacillus* species [41], suggests that there are many more *Lactobacillus* phages and plasmids that have yet to be sequenced or characterized. Further investigation of the phage communities within the vaginal and urinary tract, especially those of key bacterial species, is an essential first step in ascertaining if a biologic factor is associated with urogenital tract symptoms.

## Supporting information

S1 Table Phages identified by PHASTER in 11 urinary *L*. *jensenii* genomes. (XLSX)

S2 Table Annotations of Lu-1 sequences. (XLSX)

S3 Table List of metagenomic samples including Lv-1 and/or Lu-1 genomic sequences. (XLSX)

S4 Table Results of PCR-based identification of Lv-1 and Lu-1 sequences in *L*. *jensenii* isolate tested. (XLSX)
